# The Carers’ Needs Assessment for Dementia (CNA-D): a validation study in the Italian population

**DOI:** 10.1007/s10072-021-05285-0

**Published:** 2021-05-04

**Authors:** Milena Zucca, Elisa Rubino, Alessandro Vacca, Paola De Martino, Marcella Caglio, Andrea Marcinnó, Mario Bo, Innocenzo Rainero

**Affiliations:** 1 Department of Neuroscience “Rita Levi Montalcini”, Aging Brain and Memory Clinic, University of Torino, Via Cherasco 15, 10126 Turin, Italy; 2 Department of Neuroscience and Mental Health, A.O.U. Città della Salute e della Scienza di Torino, Turin, Italy; 3 Department of Medical Sciences, Section of Geriatrics, University of Torino, Città della Salute e della Scienza di Torino, Turin, Italy

**Keywords:** Caregiver, Dementia, Need assessment, Italian, Validation, CNA-D

## Abstract

**Background:**

Dementia has devastating consequences for families with important physical, psychological, social, and financial effects. Evaluation of caregiver’s needs may be an important step to reduce the burden of family caregivers of dementia patients. An Austrian scale, the Carers’ Needs Assessment for Dementia, is now available for measuring the caregiver’s needs. The aim of our study was to evaluate the psychometric properties of the Italian version of the CNA-D (iCNA-D).

**Methods:**

A sample of 214 voluntary caregivers of dementia patients was recruited at the Department of Neuroscience, University of Turin (Italy). All participants were administered the iCNA-D. Validity and reliability of the instrument were evaluated using Beck Depression Inventory (BDI), Beck Anxiety Inventory (BAI), Symptom Checklist-90 (SCL-90), and the Italian version of Zarit Burden Interview (I-ZBI).

**Results:**

The most common unmet need reported for the iCNA-D was “counseling and emotional support” (31.5%). This item demonstrates adequate reliability with moderate internal consistency for all “summary scores” of iCNA-D (*α* ≥ 0.75) and split-half correlation of more than 0.80 for two of them. We also found positive correlations in two out of three “summary scores” of iCNA-D and in the overall outcomes of BDI, BAI, SCL-90, and I-ZBI.

**Conclusions:**

The iCNA-D could be a valid and reliable tool for a comprehensive assessment of needs and possible social supports proposed to relatives who take care of patients with dementia. Better understanding of family caregivers’ needs could improve planning of local services and reduce caregivers’ perception of distress and burden.

## 1.Introduction

With the progressive increase of elderly population, dementia has become a dramatic health and socio-economic problem. In absence of therapeutic strategies capable of even slowing the progression of the syndrome, in 2050, there will be more than 150 million people affected with dementia worldwide [1].

During its course, dementia has devastating effects also on family members who are involved in patient’s care, usually named as “informal caregivers” [2]. It is well recognized that the increasing burden of care along with the progression of dementia has important physical, psychological, social, emotional and financial adverse effects for informal caregivers [3]. Moreover, the increasing frailty of the caregiver may result in an early patient’s institutionalization [4, 5].

It has been assumed that the evaluation of caregiver’s needs may be an important step for planning social and health services dedicated to caregivers and patients with cognitive impairment [6–8]. Van Haaster et al. [9] suggested an evaluation of needs divided into three different levels of assessment: (1) the problems experienced by patient or caregiver, (2) the interventions necessary to reduce these problems and (3) the services required to make these interventions available.

To the best of our knowledge, the Carers’ Needs Assessment for Dementia (CNA-D) [10] is the only scale psychometrically robust [11] recommended for research purposes for planning local services [12]. This is in accordance with the suggestions of Van Haaster et al. [9] and, in line with literature [13], involves the perspective of both carers and experts for a more comprehensive assessment of unmet needs of caregivers.

The CNA-D was used by Wancata et al. [10] for evaluation of needs in informal caregivers of patients with a severe cognitive impairment due to Alzheimer’s disease. These authors [10] found that the most important problems experienced by caregivers of patients with a severe cognitive impairment were disappointment caused by the illness and concerns about the patient’s future (68.9%). Furthermore, these authors have also highlighted that a number of problems reported by the interviewed caregivers were often not satisfied at all by available supports and services. This result, in line with literature [14], is remarkably interesting because it is recognized that a higher number of unmet needs can increase levels of psychological distress, depression, anxiety and physical impairments [3, 15, 16].

To date there has not been any validation of this scale on Italian population, yet. The aim of this research is to validate the CNA-D in an Italian population of caregivers of patients affected by the most common forms of dementias and with different degrees of cognitive and functional abilities impairment.

## 2.Methods

### 2.1.Sample and setting

Two hundred and fourteen caregivers (M/F=72/142; mean age ± SD= 64 ±13 years) of patients with a previously established primary diagnosis of major neurodegenerative disorder (i.e., Alzheimer’s disease (AD), frontotemporal dementia (FTD), vascular dementia (VaD), and dementia with Lewy bodies (DLB)) were recruited on voluntary basis at the Department of Neuroscience, University of Torino (Italy). Participants were included in the study if they were the principal caregiver of a patient with a primary diagnosis of dementia. Relatives were excluded from the study if they had a history of severe cognitive impairment or mental illness. The family caregivers’ interviews were conducted by a trained psychologist in different settings, including outpatient clinics, day centers, and inpatient and residential care. The relatives were interviewed in person, by phone or via Skype platform.

### 2.2.Measurements and instruments

In our study, we considered only the demographic and clinical data acquired in no more than three months before the date of the interview. Degree of cognitive impairment was assessed according to the most recent patient’s raw scores at the Italian version of Mini Mental State Examination (MMSE) [17], at the Clinical Dementia Rating Scale (CDR) [18] or at the Short Portable Mental Status Questionnaire (SPMSQ) [19]. Patients were then divided into three subgroups: (1) patients with mild cognitive deficit; (2) patients with a moderate cognitive deficit, and (3) patients with a severe cognitive deficit [20].

Functional status of patients was assessed with the Activities of Daily Living scale (ADL) [21] and the Instrumental Activities of Daily Living scale (IADL) [22] that were administered to caregivers during the interview*.* Patients were divided again in three subgroups: 1) patients completely autonomous or only partially dependent; 2) moderately dependent patients and 3) patients completely dependent [20].

Caregivers were interviewed with an Italian version of CNA-D, a semi-structured questionnaire that investigates the problems found by each subject in the caring of patient in the three months before the interview. The CNA-D includes a total of 20 problem areas: 18 predefined problem areas in the main interview related to the principal social, emotional, and practical needs perceived from caregivers, an additional 19^th^ area for the assessment of possible additional problems experienced by caregiver (not mentioned in the main interview).

A separate problem area is used only in case of a recent onset of the disease. Problem severity is rated on a three-point scale: no or mild problem; moderate problem; serious problem. For each problem with a severity degree between moderate to serious, referred by the caregiver and/or the expert, CNA-D scale offers two to six different interventions, including an additional item called “other intervention” referring to supports which are not present in the previous helps list.

Caregivers and experts rated each intervention using a five-point scale, as summarized below:

- 0 = “no need”: if the intervention was not needed and not received;
- 1 = “overprovision”: if the intervention was not needed, but received;
- 2 = “unmet need”: if intervention was needed, but not received;
- 3 = “partially met need”: if the intervention was needed and insufficiently received;
- 4 = “met need”: if the intervention was needed and sufficiently received.

Where selection of supports is concerned, the authors underline that only the most appropriate help to solve a given problem is usually rated as “needed intervention.” Occasionally, more supports can be marked as need, only if a problem area can be ameliorated with a combination of several interventions simultaneously.

The psychometric properties of the CNA-D can be viewed in the original article of Wancata et al. [10]. Authors reported an internal consistency (Cronbach’s *α* coefficient) of .70 for the “summary score” of problems, of .95 for the “summary scores” of met needs and of .88 for the “summary scores” of unmet needs according to the caregiver’s evaluation. The Cronbach’s *α* coefficient for the interviewers’ ratings, instead, were reported to be .72, .96 and .90. Furthermore, the estimation of instrument validity [10] reported a positive correlation between the total score of Zarit Burden Inventory (ZBI) [23] and the number of moderate or serious problems both marked by relatives (*r* = .683) and experts (*r* =.673). Finally, the carers’ burden was positively correlate with “summary scores” of unmet needs (carers: *r* =.523, experts: *r* =.502), and negatively associated with “summary scores” of met needs (carers: *r* = −.333, experts: *r* = − .456).

Three Italian researchers (MZ, AV, ER) independently translated the original English version of CNA-D [10] into Italian. All versions were compared to produce a draft which was translated into English by an additional researcher (PD) and finally compared with the original English version.

To test the psychometric properties of the Italian version of CNA-D, caregivers were administered specific questionnaires evaluating subjective burden degree, psychological distress and emotional situation of caregivers: the Italian version of ZBI (I-ZBI) [24], the Symptom Checklist-90-Revised (SCL-90-R) [25], the Beck Depression Inventory (BDI) [26], the Beck Anxiety Inventory (BAI) [27], and the Apathy Evaluation Scale–Clinician Version (AS) [28].

We chose these specific instruments because social burden, psychological distress and emotional status dimensions are reported to be related to the presence of a higher number of caregivers’ problems and unmet needs [29]. The validation protocol was preliminary administrated to forty caregivers of patients with dementia.

### 2.3.Data analysis

All data were analyzed using SPSS version 21.0 for Windows (IBM SPSS Statistics, Inc., Chicago, Illinois). The significance level was set at *p* < .05 for all analyses. Demographic and clinical characteristics of caregivers and patients are presented as frequencies and percentages. Cronbach’s *α* coefficient and the split-half correlation were used to calculate the relation among the “summary scores” of the iCNA-D. In detail Cronbach’s *α* coefficient evaluates internal consistency in relation with the average correlation between items, while split-half correlation, a measure of reliability, is a comparison between even and odd items of each abovementioned “summary score.” To analyze the construct validity, we performed *a* Spearman’s correlations between the three “summary scores” of the iCNA-D and the sum-score of the I-ZBI, SCL-90-R, BDI, BAI, and AS. In addition, subscales’ scores of SCL-90-R were included in the correlation analysis.

## 3.Results

### 3.1.Characteristics of the sample

The demographic and clinical characteristics of patients and caregivers are shown in Table 1. In our study, 142 (66%) of recruited caregivers were women, half spouse (104; 49%) and half children (102; 48%) of patients, while 3% of caregivers were grandchildren. The mean age of the sample was 64 ± 13 years. About 62% of relatives involved in the study lived with patients and the majority had a private home care support (68%) more often given by other family caregiver (44.1%). Mean of years schooling in our sample was 12 ± 4 years, with most of the caregivers having a high school education (42%). The majority of patients had a diagnosis of AD (61%) and a severe cognitive impairment (47%). ADL and IADL had mean values respectively of 3.62 ± 2.06 and 2.57 ± 2.61.

**Table 1:** Synopsis of the demographic and clinic characteristics of patients and caregivers

	Value and percentage (*N* (%))	Mean (±SD) years
Caregiver characteristics; *N*=214		
Sex		
Male	72 (34%)	
Female	142 (66%)	
Age		64 ±13
≥ 65 years	109 (51%)	
< 65 years	105 (49%)	
Education		12 ± 4
Primary	27(13%)	
Secondary	69 (32%)	
High	89 (42%)	
University	29 (14%)	
Relationship with patient		
Spouse	104 (49%)	
Children	102 (48%)	
Others	8 (3%)	
Care support		
No	69 (32%)	
Yes	145 (68%)	
Care for the patient by an informal caregiver	64 (44.1%)	
Care for the patient in a day center	47 (32.4%)	
Care for the patient by a formal caregiver	34 (23.4%)	
Patient characteristics; *N*=214		
Diagnosis		
AD	131 (61%)	
VaD	45 (21%)	
FTD	30 (14%)	
DLB	8 (4%)	
Cognitive status		
No or mild cognitive impairment	46 (21%)	
Moderate cognitive impairment	68 (32%)	
Severe cognitive impairment	100 (47%)	
Functional status		
ADL		3.62 ±2.06
No or mild impairment	118 (55%)	
Moderate impairment	77 (36%)	
Severe impairment	19 (9%)	
IADL		2.57 ±2.61
No or mild impairment	49 (23%)	
Moderate impairment	100 (47%)	
Severe impairment	65 (30%)	

*AD*, Alzheimer’s disease; *FTD*, frontotemporal dementia; *VaD*, vascular dementia; *DLB*, dementia with Lewy bodies; *ADL*, activities of daily living; *IADL*, instrumental activities of daily living

The percentage of the problems evaluated as moderate or serious by caregivers and interviewers are summarized in Table 2. The most frequent problem for subjects providing direct care to patients with dementia was “disappointment caused by the illness, concerns about the patient’s future” (57.9%). The interventions selected by caregivers and experts are summarized in Table 3. As reported by Wancata et al. [10], although during the interview identical interventions were presented and evaluated separately for each problem area, during the analysis similar intervention were merged to avoid redundancies.

**Table 2:** Percentages of problems evaluated as moderate or serious by caregivers and interviewers

	Carer (%)	Interviewer (%)
Lack of information about dementia	30.8	30.8
Lack of information about treatment	21.5	21.5
Lack of information about services	25.7	25.7
Financial burden	35.0	35.0
Legal issues	15.4	15.4
Disappointment caused by the illness, concerns about the patient’s future	57.5	57.9
Communication problems and conflicts with the patient	51.9	51.9
Burdened by behavioral problems of the patients	38.3	38.3
Problems caused by crises	19.2	19.6
Not enough time for oneself (including caring for the patient when the relative becomes sick)	53.3	52.8
Social isolation, conflicts within the family	36.0	36.0
Burden caused by dangerous situations	37.4	37.4
Fear of stigmatization and discrimination	12.1	12.6
Feelings of guilt, being blamed	17.3	17.8
Missing nursing skills	15.9	15.9
Difficulties concerning household tasks	37.9	37.9
Burned out or overstrained by care	45.3	45.8
Physical or psychiatric illness of the carer	45.3	46.3
Others	0.0	0.0

**Table 3:** Percentages of interventions selected by caregivers and experts as most appropriate for solving one or more problems

		No need (%)	Overprovision (%)	Unmet need (%)	Partially met need (%)	Met need (%)
Counseling and emotional support	Carer	59.4	0.1	31.5	6.0	3.0
	Interviewer	59.0	0.1	32.0	5.9	3.0
Relatives group guided by a professional	Carer	93.1	0.0	4.1	1.8	1.0
	Interviewer	93.2	0.0	4.0	1.8	1.0
Self-help group for family members	Carer	94.8	0.1	2.5	1.1	1.5
	Interviewer	94.9	0.1	2.5	1.1	1.5
Individual psychoeducation	Carer	48.5	0.0	28.3	21.4	1.7
	Interviewer	48.8	0.0	28.2	21.3	1.7
Printed information material	Carer	96.9	0.0	2.3	0.8	0.0
	Interviewer	96.9	0.0	2.3	0.8	0.0
Support from a social worker	Carer	49.3	0.0	29.0	12.2	9.5
	Interviewer	49.3	0.0	29.0	12.2	9.5
Group psychoeducation	Carer	80.4	0.0	12.3	5.2	2.1
	Interviewer	80.4	0.0	12.3	5.2	2.1
Financial compensation	Carer	44.7	1.3	17.1	23.7	13.2
	Interviewer	44.7	1.3	17.1	23.7	13.2
Temporary supervision of the patient at home	Carer	65.4	0.0	26.3	6.4	1.8
	Interviewer	66.2	0.0	25.9	6.1	1.8
Care for the patient in a day center	Carer	56.5	0.0	13.9	19.6	10.0
	Interviewer	56.8	0.0	13.6	19.6	10.1
Mobile personal care for outpatients	Carer	74.6	0.0	14.4	6.7	4.3
	Interviewer	74.3	0.0	14.7	6.7	4.3
Training of practical skills for the carer (e.g., basic nursing skills)	Carer	97.5	0.0	2.1	0.4	0.0
	Interviewer	97.1	0.0	2.1	0.4	0.4
Diagnosis or treatment of the carer by a GP	Carer	84.5	0.5	4.6	6.2	4.1
	Interviewer	84.3	0.5	5.1	6.1	4.0
Mobile nursing care for outpatients	Carer	71.1	0.0	12.0	9.5	7.4
	Interviewer	71.1	0.0	12.0	9.5	7.4
Holidays together with the patient in a specialized setting	Carer	100.0	0.0	0.0	0.0	0.0
	Interviewer	100.0	0.0	0.0	0.0	0.0
Respite care	Carer	96.4	0.0	2.7	0.9	0.0
	Interviewer	96.5	0.0	2.6	0.9	0.0
General assistance for household chores	Carer	51.2	0.0	29.6	9.9	9.3
	Interviewer	51.2	0.0	29.6	9.9	9.3
Hotline, where the carer can get advice in crises	Carer	73.2	0.0	19.5	4.9	2.4
	Interviewer	73.8	0.0	19.0	4.8	2.4
Psychotherapy	Carer	68.5	0.0	27.0	3.1	1.4
	Interviewer	68.7	0.0	26.9	3.0	1.3
Social contact service	Carer	98.7	0.0	1.3	0.0	0.0
	Interviewer	98.7	0.0	1.3	0.0	0.0
Mobile crisis service visiting the family at home	Carer	63.4	0.0	31.7	4.9	0.0
	Interviewer	59.5	0.0	35.7	4.8	0.0
Specialized services to help with specific household tasks (e.g., laundry service)	Carer	97.5	0.0	1.2	1.2	0.0
	Interviewer	97.5	0.0	1.2	1.2	0.0
Meals-on-wheels	Carer	98.8	0.0	1.2	0.0	0.0
	Interviewer	98.8	0.0	1.2	0.0	0.0
Initiating to become a legal administrator	Carer	66.7	0.0	18.2	12.1	3.0
	Interviewer	66.7	0.0	18.2	12.1	3.0

The intervention more often reported as necessary by caregivers was “financial compensation” (53.9%), while that most common unmet need, according to the caregivers and interviewers, was “counseling and emotional support” (31.5%). In our sample, relatives and experts reported that the “financial compensation” is often a partially (23.7%) or completely (13.2%) met need. Very rarely an intervention was evaluated by caregivers or interviewers as “overly given.” The mean total scores of I-ZARIT, SCL-90, BDI, BAI, and AS were 21.75 ± 18.52, 22.33 ± 20.47, 6.22 ± 5.06, 7.25 ± 6.10, and 5.49 ± 5.83, respectively.

### 3.2.Reliability and validity

Concerning the reliability analysis, Cronbach’s *α* coefficient was .79 for the “summary score” of problems, .75 for the “summary score” of met needs, and .78 for “summary score” of unmet needs reported by the caregivers. The “summary scores” of problems, of met and unmet needs according to the interviewers’ ratings generated *α* coefficients of .79, of .75, and of .77, respectively. The split-half correlation coefficient was .84 for the “summary score” of problems, .62 for the “summary score” of met needs and .86 for “summary score” of unmet needs reported by the relievers. A slit-half correlation coefficient of .84, of .62, and .85 was obtained by the “summary score” of problems, of met needs, and of unmet needs according to the experts’ evaluations.

Regarding the validity analysis the Spearman’s correlation showed a significant positive correlation between the iCNA-D “summary score” of problems and unmet needs reported by the caregivers and experts and the total score of I-ZBI (*p* < .01) and of SCL-90-R (*p* < .01). Furthermore, we also found that the iCNA-D “summary score” of problems and unmet needs indicated by the relatives and interviewers was positively related to subscales of SCL-90 (*p* < .05) and the total score of BDI (*p* < .01) and BAI (*p* < .01). Finally, the iCNA-D “summary score” of met needs was negatively associated with total score of I-ZBI (caregivers: *p* = .043, interviewers: *p* = .047) and “paranoid ideation” subscale of SCL-90 (caregivers: *p* = .012, interviewers: *p* = .011) (Table 4).

**Table 4:** Correlations of caregivers’ “summary scores” of iCNA-D with other assessment tools. *I-ZBI*, Italian version of the Zarit Burden Interview; *SCL-90-R*, Symptom Checklist 90-Revised; *BDI*, Beck Depression Inventory; *BAI*, Beck Anxiety Inventory; *AS*, Apathy Evaluation Scale

		“Summary score” of problems	“Summary scores” of met needs	“Summary scores” of unmet needs
I-ZBI (total score)	Carer	0.731^**^	− 0.138^*^	0.819^**^
	Interviewer	0.736^**^	−0.136^*^	0.828^**^
SCL-90-R Global Symptom Index	Carer	0.649^**^	0.004	0.654^**^
	Interviewer	0.659^**^	0.008	0.666^**^
SCL-90-R Somatization	Carer	0.375^**^	−0.022	0.385^**^
	Interviewer	0.385^**^	−0.019	0.397^**^
SCL-90-R Obsessive compulsive	Carer	0.449^**^	0.022	0.441^**^
	Interviewer	0.461^**^	0.025	0.449^**^
SCL-90-R Interpersonal sensitivity	Carer	0.423^**^	−0.121	0.459^**^
	Interviewer	0.430^**^	−0.116	0.468^**^
SCL-90-R Depression	Carer	0.702^**^	0.089	0.680^**^
	Interviewer	0.708^**^	0.092	0.689^**^
SCL-90-R Anxiety	Carer	0.545^**^	−0.081	0.561^**^
	Interviewer	0.552^**^	−0.077	0.572^**^
SCL-90-R Hostility	Carer	0.371^**^	−0.078	0.400^**^
	Interviewer	0.376^**^	−0.790	0.408^**^
SCL-90-R Phobic anxiety	Carer	0.154^*^	−0.078	0.186^**^
	Interviewer	0.154^*^	−0.077	0.185^**^
SCL-90-R Paranoid ideation	Carer	0.340^**^	−0.172^*^	0.387^**^
	Interviewer	0.339^**^	−0.173^*^	0.386^**^
SCL-90-R Psychoticism	Carer	0.313^**^	−0.075	0.321^**^
	Interviewer	0.314^**^	−0.077	0.321^**^
SCL-90-R Sleep disturbances	Carer	0.311^**^	−0.075	0.339^**^
	Interviewer	0.321^**^	−0.071	0.351^**^
BDI	Carer	0.614^**^	0.010	0.609^**^
	Interviewer	0.621^**^	0.012	0.616^**^
BAI	Carer	0.496^**^	−0.060	0.517^**^
	Interviewer	0.503^**^	−0.055	0.526^**^
AS	Carer	0.261^**^	0.019	0.220^**^
	Interviewer	0.258^**^	0.023	0.218^**^

^*^p < 0.05; ^**^p < 0.01

## 4.Discussion

The main purpose of our study was to evaluate reliability and validity of the Italian version of CNA-D and our data showed that iCNA-D has an adequate internal consistency, in particular for “summary score” of problems and unmet needs evaluated by both caregivers and specialists. Regarding “summary score” of met needs according to caregivers’ and experts’ evaluations, we found a Cronbach’s *α* equal to .75 in both cases, while the split-half correlation coefficient is .62. In our study, summary score of problems and unmet needs, obtained from the assessment of both caregivers and experts, positively and significantly correlates with total score of I-ZBI and BAI. A positive correlation was also found with the subscales of SCL-90. “Summary score” of met needs was, instead, negatively associated with total score of I-ZBI and “paranoid ideation” subscale of SCL-90. These results support the concurrent validity of the iCNA-D. Particularly, the existence of a strong positive correlation between total score of I-ZBI and “summary score” of problems and unmet needs confirm the hypothesis of other studies [3, 10], reporting that the presence of a greater number of problems, not solved or only partially solved, can increase the subjective burden experienced by caregivers. Furthermore, in our sample, if problems were associated with a higher number of unmet needs, it was also possible to find a major level of depression, anxiety, and other psychological symptom dimensions, such as somatization, obsessive-compulsive features, interpersonal sensitivity, hostility, phobic anxiety, paranoid ideation, and sleep disturbance. In contrast, the presence of met needs seemed to decrease the level of burden perceived by the subject and, intriguingly, seemed also to reduce psychological symptoms as projective thinking, hostility, suspicion, fear of loss of autonomy etc.

Our data are supported by other researches [15, 16, 29] in which a correlation was reported between the presence of unmet needs and an increase of subject’s psychological distress. In our study, caregivers reported the “financial compensation” as the most necessary intervention (53.9%) and simultaneously the most often partially (23.7%) or completely (13.2%) “met need,” while the lack of personal time is the second most frequently problem complained by caregivers during the interview (53.3%). Additionally, although the 68% of relievers received care support, only the 23.4% of them gets help from a formal caregiver. These findings, in line with other researches [3, 30], can be partially explained by the fact that caregivers of our sample tend not to use financial compensations to afford additional care assistance given by a formal caregiver or other similar territorial services.

In this regard, it was suggested that there are four dimensions that can affect decisions of caregivers of patients with dementia to use or refuse available services: service, personal, experiential, and relational factors [31]. In particular, it was speculated that a specific personal factor, the caregivers’ lack of acknowledgement of their unmet needs for help, can be associated to a lower utilization of services [32]. Furthermore, Zwingmann et al. [33] have recently highlighted that caregivers’ accession rate of support services can be related to the type of help offered. These suggestions further highlight the importance of having an available and specific tool for a comprehensive caregivers’ needs assessment.

The most frequently unmet need reported by carers in our study was “counseling and emotional support” (31.5%). This result is confirmed by past literature evidence [34–36] reporting that a very few informal caregivers of patients with dementia receive counseling and emotional support services. Very often the only emotional support comes from other family members and this can increase the level of relieves’ burden, negatively affecting the relationship with the patient. Interestingly, in our sample the unmet or only partially met needs have been reported much more frequently than met needs. These frequencies are in line with the findings of Wancata et al. [10]. Since several variables including clinical patients’ characteristics [37–39] can affect the caregiver’s distress, we have decided to include in our study caregivers of subjects with different diagnosis of dementias and cognitive and functional abilities impairment. We hypothesize that this choice could increase predictive capabilities of this tool and make it more usable in the future research.

Our study has some limitations that should be acknowledged. Firstly, in the “Back Translation” phase we have only involved an Italian researcher that is also a bilingual translator. “Back Translation” step is one type of validity check highlighting conceptual errors in the translation. Since the constructs assessed by the CNA-D are essentially culturally shareable, we assume that this limitation does not invalidate the linguistic validation of the instrument. Secondly, pre-test of iCNA-D was performed only by caregivers and we have not used a specific strategy (e.g., cognitive interviews) to conduct a more in-deep pre-test assessment. However, in the original validation study, Wancata et al. [10] had already extensively developed the pre-test phase on both carers and experts. Finally, socio-demographic caregivers’ features [40] and clinical patients’ characteristics [37–39] that often affect the caregiving burden were not explored widely in this study because it was not the principal aim of our research. However, future studies that will use this tool could more in-deep investigate these dimensions.

## 5.Conclusions

In our study, we evaluated the reliability and validity of the Italian version of CNA-D and we found that this scale could be a valid and reliable tool for a comprehensive assessment of needs and possible social supports proposed to caregivers of patients with dementia. Clarifying the structure of the iCNA-D has potential social and health implications. Better understanding of needs of relatives that assist patients with dementia could improve targeted intervention programs for planning specific local services, with a consecutive reduction of burden and an increase of quality of life for caregivers.
